# Dietary patterns and associations with metabolic risk factors for non-communicable disease

**DOI:** 10.1038/s41598-023-47548-0

**Published:** 2023-11-29

**Authors:** Tilahun Tewabe Alamnia, Ginny M. Sargent, Matthew Kelly

**Affiliations:** 1 National Centre for Epidemiology and Population Health, Australian National University, 62 Mills RD, Acton ACT 2601, Canberra, Australia; 2 College of Medical and Health Sciences, Bahir Dar University, Bahir Dar, Ethiopia

**Keywords:** Health care, Medical research, Risk factors

## Abstract

Unhealthy dietary habit is a major contributor to the burden of non-communicable diseases such as cardiovascular diseases, diabetes, and hypertension, especially the increased burden in low- and middle-income countries. Evidence of the association between specific dietary patterns and health outcomes is scarce in sub–Saharan African countries. This study aimed to identify principal dietary patterns and evaluate associations with metabolic risk factors including hypertension, overweight/obesity, and abdominal obesity in Northwest Ethiopia. A community-based cross-sectional survey was conducted among adults in Bahir Dar, Northwest Ethiopia, from 10 May 2021 to 20 June 2021. Dietary intake was collected using a validated food frequency questionnaire. Anthropometric (weight, height, hip/waist circumference) and blood pressure measurements were performed using standardized tools. Principal component analysis was conducted to derive dietary patterns. Chi-square and logistic regression analyses were used to examine the association between dietary patterns and metabolic risk factors and with sociodemographic and individual risk factors. This study derives two types of dietary patterns: ‘westernized’ dietary pattern, which is positively correlated with consumption of meat, dairy, fast foods, alcohol, fish, sweet/sugary foods, and fruits, and ‘traditional’ dietary pattern, which is positively correlated with intake of cereals, vegetables, legumes, roots/tubers, coffee, and oils. The prevalence of hypertension was significantly lower in adults with higher quantiles of westernized dietary pattern (AOR = 0.28, 95% CI 0.13 to 0.60; *p* < 0.01; quantile three); and (AOR = 0.35, 95% CI 0.17, 0.75; *p* < 0.01; quantile four). Younger, married, and middle-income adults were associated with the highest quantile of the westernized pattern. Being females and having middle income associated with the highest quantile of traditional dietary patterns (*p* < 0.05). This study suggested two types of dietary patterns, westernized and traditional, among adults in Northwest Ethiopia and revealed a significant association with metabolic risk factors like hypertension. Identifying the main dietary patterns in the population could be informative to consider local-based dietary recommendations and interventions to reduce metabolic risk factors.

## Introduction

Metabolic risk factors, such as high body mass index, abdominal obesity, and hypertension have been widely recognized as significant factors to the increasing burden of non-communicable diseases (NCDs)^1^. They cause metabolic disturbances that increase the likelihood of chronic diseases, such as diabetes, cardiovascular diseases (CVDs), stroke, and kidney diseases^2^. It is estimated that over one billion people, one in four men and one in five women, are hypertensive, and more than one in three adults are overweight or obese worldwide^3,4^. Metabolic risk factors, which are associated with dietary habits, are highly prevalent in developing countries, serving as the major driver for the growing burden of NCDs in these settings^5–7^.

Food consumption and dietary patterns are the results of various interrelated phenomena, the specifics of which vary greatly by region: geography, environment, agriculture, culture/tradition, societal beliefs and norms, civilization, and globalization^8,9^. Studies show that food security issues in a population, which encompasses food availability, access, utilization, and stability^10^, can lead to changes in dietary patterns, such as consuming more processed and high-calorie foods and a lower intake of fruit and vegetable and less diversified foods^11^. This can also lead to inadequate intake of essential nutrients, such as protein, vitamins, and minerals which can contribute to the development of metabolic risk factors such as obesity, dyslipidaemia, and hypertension, particularly in low-income populations^12–14^.

Previous studies on dietary predictors of metabolic risk factors have been mainly focused on the effect of single nutrients and foods, such as salt and fat intake^15^. The study of dietary pattern analysis, which considers food consumption as a whole rather than a single nutrient-based analysis for a more effective nutrition intervention, has recently emerged as an alternative method to explore associations between dietary habits and health outcomes^16^. For example, dietary pattern analyses in Mexican adolescents and Lebanese adults show that westernized dietary patterns, characterized by higher intakes of packaged and fast foods, are associated with increased rates of hypertension and body mass index (BMI)^17,18^. In developing countries like Ethiopia, suboptimal dietary consumptions, early-age chronic undernutrition caused by dietary insufficiency, and the growing burden of overweight/obesity in adults are overwhelming the national healthcare response^19,20^. Previous studies in Ethiopia identified several sociodemographic predictors of metabolic risk factors, including gender, marital status, residence, occupational status, income, and with individual risk factors such as inadequate fruit/vegetables intake, salt intake, physical inactivity, alcohol consumption, tobacco use, and khat chewing^21–25^. However, little is known about the association between dietary patterns and metabolic risk factors in the population.

Considering the above research gaps, this study aims to (1) derive the dietary patterns of adults in Bahir Dar Northwest Ethiopia using a principal component analysis (PCA) and (2) examine the association between dietary patterns and metabolic risk factors. The results of this study can inform policymakers, healthcare providers, and stakeholders to integrate dietary packages in the prevention and control of NCDs.

## Methods

### Study design, setting, and participants

A community-based cross-sectional survey was conducted between May and June 2021. All study procedures were performed and reported in accordance with the STROBE (Strengthening the Reporting of Observational Studies in Epidemiology) guidelines (S1 appendix).

This study was conducted in Bahir Dar, Northwest Ethiopia. Bahir Dar is the capital city of Amhara regional state, which is located 565 km away from the country’s capital city, Addis Ababa. The town has six sub-cities and 17 administrative units called *kebele* in the local language. Based on the Central Statistical Agency report, the town’s total population was 221,991 in 2007^26^, and is currently estimated to be around half a million^27^. With regard to healthcare facilities, Bahir Dar has one referral hospital, two district-level public hospitals, four private hospitals, and ten primary healthcare centers.

This study is based on information collected from 423 adult participants. Which is part of a community-based survey of non-communicable diseases and risk factors among adults in Northwest Ethiopia reported elsewhere^28^. This sample size was calculated using a single population proportion formula (Za/_2_^2^ p(1-p)/d^2^) with the following assumptions: prevalence (p) of 50%, the standard normal distribution value at 95% confidence level (Z) = 1.96, a margin of error (d) = 5%, and a 10% estimated non-response rate. There was no similar study in the study area to inform the sample size determination, thus 50% prevalence rate was used to maximize the sample size estimation. Adult residents who had lived in the study area for at least six months and aged between 18 and 65 years were eligible to participate in this study. Participants were recruited from residential houses in each sub-city using a systematic random sampling technique. The total number of formally registered residential houses in each sub-city was obtained from administrative offices (N = 12,000). The research team randomly started at a house in a neighborhood then proceeded using a fixed interval (n ≈ 28) to select the next study participant, which was estimated based on the total number of formally registered residential houses (N = 12,000) and the calculated sample size (n = 423). Data collectors (nurses) received two days of training on how to approach study participants, provide relevant information, take informed consent, perform interviews and measurements, and managing the collected data. Measurement tools and techniques were pretested in 30 participants in a similar setting near Bahir Dar kebele-8 before the actual data collection time to check the clarity of questionnaires, data collection techniques, and participant response rate. The principal investigator closely supervised and monitored the process throughout the data collection period.

### Dietary intake assessment

Adults’ nutrition assessment was performed using a validated food frequency questionnaire (FFQ). The questionnaire was adopted from a previous study that validated the dietary habits of adults in Ethiopia^29^. The FFQ includes fourteen food groups: cereals, roots and tubers, vegetables, fruits, meat, egg, fish, legumes, dairy, oil/fat, sweets/sugar, coffee/tea, alcohol, and fast foods. Each food group consists of food items read to participants during interviews. For example, the cereal group consists of food items such as local foods injera, bread, rice, noodles, biscuits, or other foods made from millet, sorghum, maize, rice, wheat, or any other locally available grain. Using the FFQ, participants were asked ‘During the past one month how often did you usually consume the following food groups?’ There were eight response categories: every day, 4–6 days per week, 2–3 days per week, one day per week, two days per month, one per month, less than one day per month, and not at all.

### Metabolic risks measures

Physical measurements such as weight, height, hip/waist circumference, and blood pressure of participants were performed using standardized measuring tools. Weight of participants was measured using Seca 874 Portable Flat Platform Weight Scales. Participants were asked to take off heavy clothes and shoes during weight measurement. Wight measures were recorded in in kilograms (kg) in one decimal point. Height of participants was measured using fixed non-bending wooden meters. During height measurement, participants were asked to take off shoes and stand upright with heels, knees, hip, shoulder, and occiput touching against the wall and gaze straight horizontally. Height measures were recorded in centimeters (cm). Waist/hip circumference were measured using non-stretchable meters. Waist circumference was measured at the midpoint of the end of ribs and iliac crest, and hip circumference was measured by passing the symphysis pubis anteriorly and the largest part of the buttock posteriorly. Waist/hip circumference measures were recorded in centimeters. Blood pressure of participants was measured using aneroid sphygmomanometers and stethoscope. Systolic and diastolic blood pressure were recorded in millimeter mercury (mmHg). Blood pressure measures were performed twice, and the average was taken to determine participants blood pressure status. Hypertension was defined when the average blood pressure measurements was ≥ 140/90 mmHg. We calculated participants’ BMI, and values 25–30 kg/m^2^ are considered overweight and ≥ 30 obesity^30^. We also calculated the hip-to-waist circumference (WHR) ratio, with values ≥ 0.85 for females and ≥ 0.90 for men indicating the presence of abdominal obesity^31^.

### Other variables collected and classifications

Sociodemographic and individual risk factors were collected to examine their association with dietary patterns and metabolic risk factors. Demographic: Age (continuous scale), sex (male, female), marital status (single, married, and others); Socioeconomic: Educational status (primary and lower, secondary level, post-secondary), occupational status (securely employed such as government or private organization employed; insecure jobs such as daily work, factory, building, garment, garage, agriculture, driver, and merchant; and unemployed), and income (continuous scale); Individual risk factors: Current alcohol consumption (yes, no), current tobacco use (yes, no), current khat chewing (yes, no), and physical activity (active, inactive).

### Statistical analyses

The collected data were coded and entered using Epi Data software version 3.1 and exported to SPSS (statistical package for social sciences) version 28 for descriptive (frequencies and percentage) and inferential analyses (Chi-square tests, PCA, and logistic regression). We first performed PCA to derive dietary patterns in the population. The Kaiser-Mayer-Olkin (KMO) and Barrel sphericity tests were performed for sampling adequacy. These tests showed that the sampling adequacy was generally good (0.61, *p* < 0.01). The PCA was performed using Promax rotation, and factors with eigenvalues above one, cross-matched with parallel analysis, were first retained. However, later, after assessing the relevancies of the factors we determined to retain two factors that best describe the principal dietary patterns of the population. Factor loading values greater than 0.2 were retained, considering that values below 0.2 do not significantly affect the variability of the outcome. The retained factors were labeled (named) consistent with previous literature and food culture/taboos in the study area^32–34^. We computed quantiles of the identified dietary pattern scores to examine variations by sociodemographic and individual lifestyle factors. We examined the association between identified dietary patterns and metabolic risk factors (hypertension, overweight/obesity, and abdominal obesity) using logistic regression analysis. We first performed bivariable analysis to examine associations between demographic, socioeconomic, and individual risk factors with metabolic risk factors. Variables that showed associations (*p* < 0.25) in the bivariable analysis and quantiles of the identified dietary pattern scores were adjusted in the multivariable logistic regression analysis to identify significant predictors of metabolic risk factors. We examined the presence of collinearity among the variables; a variance inflation factor (VIF) of less than three was achieved for all variables, indicating the absence of collinearity. The final model was checked for significant Omnibus tests of model coefficients (*p* < 0.05) and non-significant Hosmer–Lemeshow goodness fit test (*p* > 0.05). Statistical significance was declared at a *p* < 0.05 for all parameters.

### Ethics approval and consent to participate

All the study methods and procedures were conducted according to the Helsinki Declarations. This study received ethics approval from the Australian National University Human Research Ethics Committee (protocol number 2020/558) and locally by the Amhara Public Health Institute Research Ethics Committee Bahir Dar, Ethiopia (protocol number 1/10,523). All participants provided informed consent to be eligible to participate in this study.

## Results

### Study participants’ characteristics

Table 1 summarizes the sociodemographic characteristics and individual risk factors of study participants. Four hundred fifteen (98.1%) adults provided dietary data in this study. The mean age of participants was 35.6 ± 12.6 years; 54.7% were females; 56.4% were married; 54.5% were post-secondary level educated; and 22.2% were securely employed. Concerning individual risk factors: 37.9% had insufficient physical activity, 95.7% had inadequate fruit/vegetable intake, 53.0% consumed alcohol, 1.9% used tobacco, and 5.0% chewed khat. The results of physical measurements show that the average BMI of adults was 23.7 ± 4.1 kg/m^2^, WHR was 0.92 ± 0.08, systolic blood pressure was 117.4 ± 13.2 mmHg, and diastolic blood pressure was 74.5 ± 10.2 mmHg. The prevalence of abdominal obesity (by WHR) was 67.4%, overweight and obesity (by BMI) was 29.6%: 21.3% were overweight and 8.3% obese; and hypertension prevalence was 20.7%. The prevalence of hypertension, overweight/obesity, and abdominal obesity varies across socioeconomic and lifestyle characteristics of adults.

**Table 1:** Socio-demographic, anthropometric, and lifestyle characteristics of study participants.

Variables	Variable category	Total n (%)	Hypertension n (%)	*P*-value	Overweight/obesity (BMI) n (%)	*P*-value	Abdominal obesity (WHR) n (%)	*P*-value
Sex	Male	189 (45.3)	41 (21.8)	0.62	46 (24.5)	0.03	104 (56.5)	0.01
	Female	228 (54.7)	45 (19.8)		76 (33.9)		171 (76.3)	
Age group	18–30	189 (45.3)	13 (6.9)	< 0.01	35 (18.6)	< 0.01	101 (54.3)	< 0.01
	31–44	146 (35.0)	24 (16.6)		52 (36.6)		102 (72.3)	
	46–65	82 (19.7)	49 (59.8)		35 (42.7)		72 (88.9)	
Marital status	Married	235 (56.4)	52 (22.3)	0.01	81 (35.1)	0.01	179 (77.8)	0.01
	Others^1^	47 (11.3)	24 (51.1)		25 (54.3)		40 (87.0)	
	Single	135 (32.4)	10 (7.4)		16 (11.9)		56 (42.4)	
Education	Primary/lower	94 (22.5)	36 (38.7)	0.01	39 (42.4)	0.01	76 (82.6)	0.01
	Secondary	96 (23.0)	14 (14.6)		25 (26.0)		61 (64.2)	
	Post-secondary	227 (54.4)	36 (15.9)		58 (25.9)		138 (62.4)	
Occupation	Securely employed	137 (32.9)	27 (19.9)	0.32	32 (23.5)	< 0.01	86 (63.7)	0.01
	Insecure jobs^2^	121 (29.0)	25 (20.7)		30 (24.8)		66 (55.5)	
	Unemployed	159 (38.1)	34 (21.5)		60 (38.7)		123 (79.9)	
Income	00–2250	53 (13.7)	13 (24.5)	0.62	12 (22.6)	0.47	31 (59.6)	0.36
	2251–5000	126 (32.6)	23 (18.3)		37 (29.6)		86 (69.4)	
	5001 +	208 (53.7)	44 (21.3)		64 (31.2)		142 (69.6)	
Salt intake	Normal	51 (12.2)	15 (29.4)	0.10	16 (32.7)	0.62	35 (70.0)	0.68
	High	366 (87.8)	71 (19.5)		106 (29.2)		240 (67.0)	
Physical inactivity	Low	158 (37.9)	32 (20.4)	0.89	53 (34.2)	0.11	113 (72.4)	0.09
	Active	259 (62.1)	54 (20.9)		69 (26.8)		162 (64.3)	
Alcohol use	No	196 (47.0)	49 (25.0)	0.04	69 (35.6)	0.01	133 (69.6)	0.36
	Yes	221 (53.0)	37 (16.9)		53 (24.3)		142 (65.4)	
Tobacco use	No	409 (98.1)	84 (20.6)	–	121 (30.0)	–	270 (67.5)	0.76
	Yes	8 (1.9)	2 (25.0)		1 (12.5)		5 (62.5)	
Khat chewing	No	396 (95.0)	79 (20.1)	0.14	114 (29.2)	0.38	260 (67.0)	0.46
	Yes	21 (5.0)	7 (33.3)		8 (38.1)		15 (75.0)	

n (%), number and percent; ^1^other marital status (living together but not married, separated, divorced, and widowed; ^2^insecure jobs (daily work, factory, building, garment, garage, agriculture, driver, and merchant); n, number; and *p*-values are from chi-square tests.

### Dietary patterns

**Figure 1:**
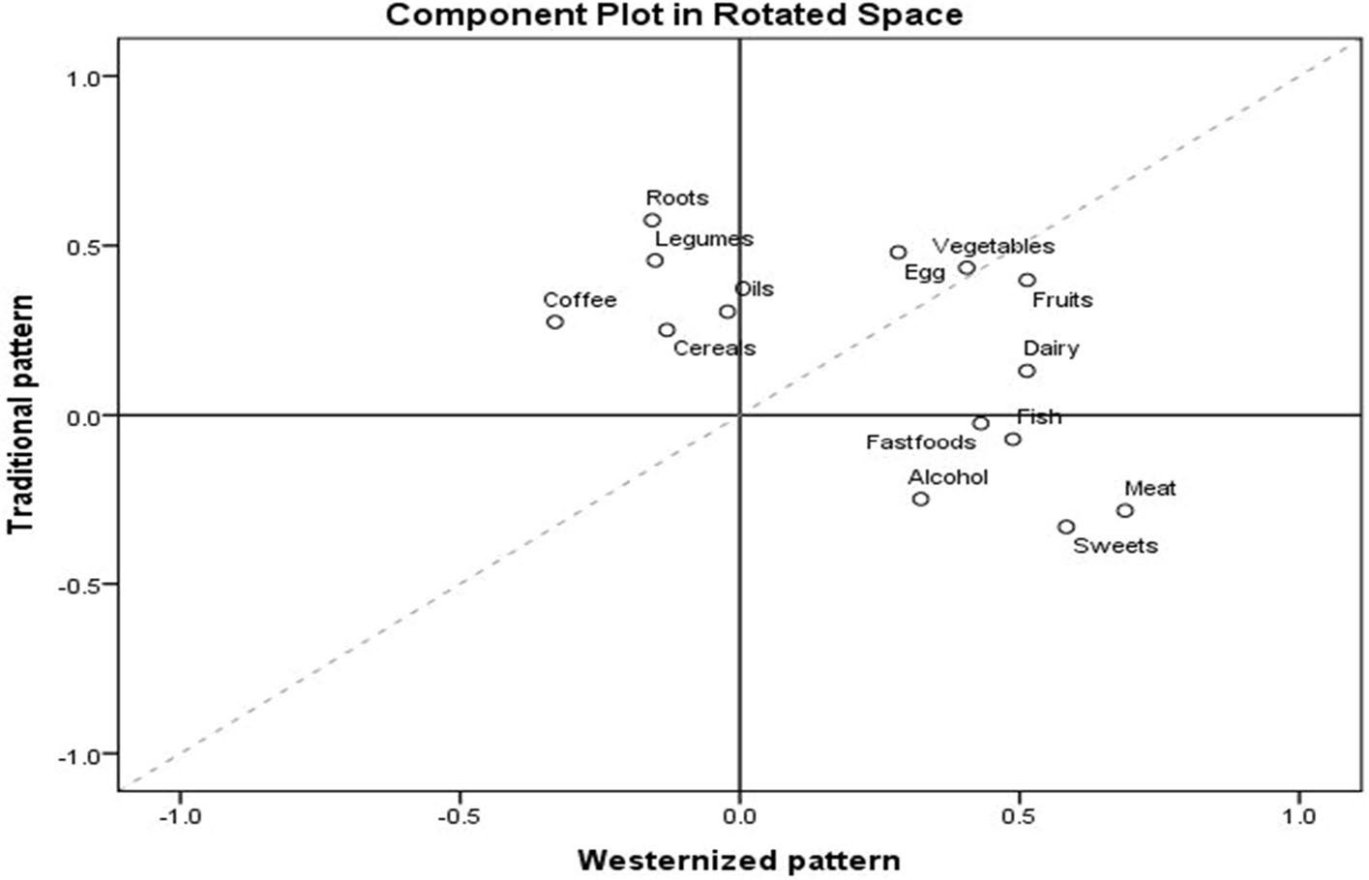
Loading component plot for westernized pattern (factor 1) and traditional pattern (factor 2).

Table 2 and Fig. 1 shows the dietary patterns of adults, and Table 3 summarises quantiles of the dietary pattern scores by socioeconomic and individual risk factors. In line with previous literature and contextual food culture, the derived factors were labeled as ‘westernized pattern’ and ‘traditional pattern.’

**Table 2:** Factor loadings of dietary patterns identified among adults in Bahir Dar.

Food groups	Median intake	Mean days per week	Dietary pattern factor loadings	
			Westernized pattern	Traditional pattern
Cereals (any local foods, injera, bread, rice, noodles, biscuits, or any other foods made from millet, sorghum, maize, rice, wheat, or any other locally available grain)	7 days per week	6.8 ± 0.9		0.25
Roots and tubers (any potatoes, kocho, carrot, beet root, yams, manioc, cassava, or any other foods made from roots or tubers)	2–3 days per week	2.4 ± 1.6		0.57
Any vegetable (like green leafy vegetables, papaya, tomato, cauliflower, cabbage, beans etc.)	2–3 days per week	2.7 ± 1.8	0.41	0.43
Fruits (like banana, mango, avocado, apple, orange, pineapple etc.)	2–3 days per week	2.4 ± 1.6	0.51	0.40
Meat (any beef, pork, lamb, goat, chicken, liver, kidney, heart, or other organ meats)	1 day per week	1.9 ± 2.3	0.69	− 0.28
Eggs (any food prepared with eggs)	2–3 days per week	2.4 ± 2.1	0.28	0.48
Any fresh or dried fish or shellfish	< 1 day per month	0.6 ± 1.5	0.49	
Legumes (Any foods made from beans, peas, lentils, or nuts)	1 day per week	2.3 ± 2.2		0.46
Dairy (any cheese, yogurt, milk, or other milk products)	2–3 days per week	2.6 ± 2.5	0.51	
Oil and fat (Any foods made with oil, fat, or butter)	7 days per week	4.7 ± 3.0		0.31
Sweets and sugar (any sugar or honey or jam)	1 day per week	3.0 ± 3.1	0.58	− 0.33
Coffee and tea (any other foods, such as condiments, coffee, tea)	7 days per week	6.1 ± 2.0	**−** 0.33	0.27
Alcoholic drinks (beer, tella, tej, arake, borde)	< 1 day per month	0.8 ± 1.6	0.32	− 0.25
Fast food and pastry (burger, pizza, cakes)	< 1 day per month	0.5 ± 1.3	0.43	

Factor loadings were obtained using Promax rotation in the PCA; the main food groups are written first and food items in the group are listed in brackets; and the mean number of days was calculated by coding the response categories into average days per week.

**Table 3:** Quantiles of the dietary patterns by participants’ sociodemographic and individual risk factors.

Variables	Variable category	Westernized pattern				*p*-value	Traditional pattern				*p*-value
		Q1	Q2	Q3	Q4		Q1	Q2	Q3	Q4	
Age	18–30	39 (20.7)	45 (23.9)	54 (28.7)	50 (26.6)	0.01	51 (27.0)	53 (28.0)	47 (24.9)	38 (20.1)	0.09
	31–45	32 (22.2)	37 (25.7)	34 (23.6)	41 (28.5)		29 (20.1)	34 (23.6)	33 (22.9)	48 (33.3)	
	46–65	33 (40.2)	21 (25.6)	16 (19.5)	12 (14.6)		23 (28.4)	17 (21.0)	24 (29.6)	17 (21.0)	
Sex	Male	51 (27.1)	47 (25.0)	44 (23.4)	46 (24.5)	0.80	62 (33.2)	43 (23.0)	50 (26.7)	32 (17.1)	0.00
	Female	53 (23.5)	56 (24.8)	60 (26.5)	57 (25.2)		41 (18.1)	61 (26.9)	54 (23.8)	71 (31.3)	
Marital status	Married	46 (19.8)	60 (25.9)	61 (26.3)	65 (28.0)	0.03	52 (22.4)	51 (22.0)	56 (24.1)	73 (31.5)	0.01
	Others	20 (42.6)	12 (25.5)	9 (19.1)	6 (12.8)		10 (21.3)	14 (29.8)	11 (23.4)	12 (25.5)	
	Single	38 (28.1)	31 (23.0)	34 (25.2)	32 (23.7)		41 (30.4)	39 (28.9)	37 (27.4)	18 (13.3)	
Education	Primary/lower	29 (31.2)	28 (30.1)	20 (21.5)	16 (17.2)	0.14	22 (23.7)	22 (23.7)	22 (23.7)	27 (29.0)	0.26
	Secondary	25 (26.0)	25 (26.0)	19 (19.8)	27 (28.1)		26 (27.1)	32 (33.3)	22 (22.9)	16 (16.7)	
	Post-secondary	50 (22.2)	50 (22.2)	65 (28.9)	60 (26.7)		55 (24.4)	50 (22.2)	60 (26.7)	60 (26.7)	
Occupation	Securely employed	29 (21.3)	34 (25.0)	40 (29.4)	33 (24.3)	0.14	31 (22.8)	35 (25.7)	34 (25.0)	36 (26.5)	0.48
	Insecure jobs	41 (33.9)	29 (24.0)	21 (17.4)	30 (24.8)		25 (20.7)	30 (24.8)	37 (30.6)	29 (24.0)	
	Unemployed	34 (21.7)	40 (25.5)	43 (27.4)	40 (25.5)		47 (29.9)	39 (24.8)	33 (21.0)	38 (24.2)	
Income	Very low	18 (34.6)	15 (28.8)	9 (17.3)	10 (19.2)	0.04	21 (40.4)	13 (25.0)	10 (19.2)	8 (15.4)	0.01
	Low	37 (29.6)	33 (26.4)	29 (23.2)	26 (20.8)		37 (29.8)	39 (31.5)	26 (21.0)	22 (17.7)	
	Middle	39 (18.8)	46 (22.2)	60 (29.0)	62 (30.0)		39 (18.8)	45 (21.6)	61 (29.3)	63 (30.3)	
Physical activity	Active	68 (26.4)	59 (22.9)	61 (23.6)	70 (27.1)	0.32	65 (25.1)	62 (23.9)	69 (26.6)	63 (24.3)	0.77
	Not	36 (23.1)	44 (28.2)	43 (27.6)	33 (21.2)		38 (24.5)	42 (27.1)	35 (22.6)	40 (25.8)	
Tobacco use	Yes	2 (25.0)	3 (37.5)	2 (25.0)	1 (12.5)	–	1 (12.5)	4 (50.0)	1 (12.5)	2 (25.0)	–
	No	102 (25.1)	100 (24.6)	102(25.1)	102 (25.1)		102 (25.1)	100 (24.6)	103 (25.4)	101 (24.9)	
Khat chewing	Yes	3 (14.3)	8 (38.1)	6 (28.6)	4 (19.0)	0.39	4 (19.0)	6 (28.6)	4 (19.0)	7 (33.3)	0.71
	No	101 (25.7)	95 (24.2)	98 (24.9)	99 (25.2)		99 (25.2)	98 (24.9)	100 (25.4)	96 (24.4)	
WHR	High risk	70 (25.7)	74 (27.2)	61 (22.4)	67 (24.6)	0.19	68 (25.0)	70 (25.7)	64 (23.5)	70 (25.7)	0.80
	Low risk	32 (24.1)	26 (19.5)	41 (30.8)	34 (25.6)		33 (24.8)	32 (24.1)	37 (27.8)	31 (23.3)	
BMI	Overweight/obesity	36 (30.0)	26 (21.7)	29 (24.2)	29 (24.2)	0.53	34 (28.3)	31 (25.8)	22 (18.3)	33 (27.5)	0.23
	Normal	68 (23.5)	76 (26.3)	73 (25.3)	72 (24.9)		68 (23.5)	73 (25.3)	80 (27.70	68 (23.5)	
Hypertension	Yes	34 (39.5)	25 (29.1)	13 (15.1)	14 (16.3)	< 0.01	24 (28.2)	14 (16.5)	22 (25.9)	25 (29.4)	0.20
	No	70 (21.5)	78 (23.9)	91 (27.9)	87 (26.7)		79 (24.2)	90 (27.5)	80 (24.5)	78 (23.9)	

*p*-values are from chi-square tests.

The westernized pattern (factor 1) was characterized by frequent consumption of fruits, meat, dairy foods, fast foods/pastry foods, alcoholic drinks, fish-based foods, and sweet/sugar foods. The highest quantile of the westernized dietary pattern, compared to the lowest quantile (Q1), showed associations with younger age, being married, and good income of adults (*p* < 0.05).

Higher loadings of food groups such as cereals, vegetables, roots and tubers, legumes, roots/tubers, eggs, oils, and coffee and tea characterized the traditional pattern (factor 2). The highest quantile of traditional dietary patterns was associated with older age, marital status, and lower income of adults (*p* < 0.05). See supplementary Tables 1 and 2.

### Associations between dietary patterns and metabolic risk factors

Table 4 presents the association between consumption of the identified dietary patterns and metabolic risk factors (hypertension, overweight/obesity, and abdominal obesity). The prevalence of hypertension was associated with the consumption of westernized dietary patterns in the bivariable logistic regression analysis (*p* < 0.01). However, other metabolic risk factors, such as overweight/obesity and abdominal obesity, did not show significant association with the derived dietary patterns. There was a significant difference in hypertension prevalence among quantiles of westernized dietary pattern scores in the final multivariate logistic regression analysis. Adults in the third quantile of the westernized dietary pattern were 72% less likely to have hypertension than adults in the first quantile of the westernized dietary pattern scores (AOR = 0.28, 95% CI 0.13 to 0.60); and adults in the fourth quantile of westernized patterns were 65% less likely to have hypertension than adults in the first quantile (AOR = 0.35, 95% CI 0.17, 0.75; *p* < 0.01) (Table 4).

**Table 4:** The association between metabolic risk factors and dietary patterns in the logistic regression analysis.

Quantiles	Hypertension (yes/no)	Crude model		Adjusted model 1		Adjusted model 2	
Quantile 1	34/70	Ref	*p*-value	Ref	*p*-value	Ref	*p*-value
Westernized dietary pattern scores and hypertension^a^							
Quantile 2	25/78	0.66 (0.36,1.21)	0.18	0.82 (0.40,1.68)	0.58	0.63 (0.32,1.22)	0.17
Quantile 3	13/91	0.29 (0.14, 0.60)	< 0.01	0.35 (0.15, 0.80)	0.01	0.28 (0.13, 0.60)	< 0.01
Quantile 4	14/87	0.33 (0.16, 0.67)	< 0.02	0.46 (0.21,1.05)	0.06	0.35 (0.17, 0.75)	< 0.01

Quantile 1	24/79	Ref	*p*-value	Ref	*p*-value	Ref	*p*-value
Traditional dietary pattern and hypertension^a^							
Quantile 2	14/90	0.51 (0.25, 1.06)	0.07	0.48 (0.21, 1.13)	0.09	0.43 (0.20, 0.94)	0.04
Quantile 3	22/80	0.91 (0.47, 1.75)	0.76	0.79 (0.37,1.70)	0.54	0.84 (0.42,1.71)	0.63
Quantile 4	25/78	1.06 (0.56, 2.00)	0.87	1.05 (0.49, 2.25)	0.89	0.80 (0.40,1.60)	0.53

Quantile 1	36/68	Ref	*p*-value	Ref	*p*-value	Ref	*p*-value
Westernized dietary pattern and overweight/obesity^b^							
Quantile 2	26/76	0.65 (.35,1.18)	0.15	0.61 (0.32, 1.16)	0.13	0.61 (0.32, 1.18)	0.14
Quantile 3	29/73	0.75 (.42, 1.35)	0.34	0.78 (0.41, 1.49)	0.45	0.82 (0.42, 1.58)	0.55
Quantile 4	29/72	0.76 (.42, 1.37)	0.36	0.79 (0.41, 1.50)	0.46	0.79 (0.41, 1.52)	0.48

Quantile 1	34/68	Ref	*p*-value	Ref	*p*-value	Ref	*p*-value
Traditional dietary pattern and overweight/obesity^b^							
Quantile 2	31/73	0.85 (0.47, 1.53)	0.59	0.77 (0.41, 1.46)	0.43	0.77 (0.40, 1.46)	0.43
Quantile 3	22/80	0.55 (0.29, 1.03)	0.06	0.48 (0.24, 0.93)	0.03	0.46 (0.23, 0.91)	0.03
Quantile 4	33/68	0.97 (0.54, 1.74)	0.92	0.69 (0.36, 1.33)	0.27	0.66 (0.34, 1.27)	0.27

Quantile 1	70/32	Ref	*p*-value	Ref	*p*-value	Ref	*p*-value
Westernized dietary pattern and abdominal obesity^b^							
Quantile 2	74/26	1.30 (0.71, 2.40)	0.40	1.19 (0.61, 2.34)	0.61	1.31 (0.66, 2.61)	0.44
Quantile 3	61/41	0.68 (0.38, 1.21)	0.19	0.55 (0.28, 1.06)	0.07	0.61 (0.31, 1.20)	0.15
Quantile 4	67/34	0.90 (0.50, 1.62)	0.73	0.80 (0.42, 1.54)	0.51	0.91 (0.47, 1.78)	0.79

Quantile 1	68/33	Ref	*p*-value	Ref	*p*-value	Ref	*p*-value
Traditional dietary pattern and abdominal obesity^b^							
Quantile 2	70/32	1.06 (0.59, 1.91)	0.84	0.89 (0.46, 1.73)	0.73	0.89 (0.45, 1.74)	0.73
Quantile 3	64/37	0.84 (0.47, 1.50)	0.55	0.70 (0.36, 1.36)	0.29	0.65 (0.33, 1.29)	0.22
Quantile 4	70/31	1.10 (0.61, 1.98)	0.76	0.58 (0.29, 1.16)	0.13	0.58 (0.28, 1.18)	0.13

^a^Model 1 adjusted for: age, marital status, educational status, and khat chewing; Model 2 adjusted for: marital status, educational status, and kchat chewing. ^b^Model 1 adjusted for sex, marital status, educational status, occupational status, and physical activity level; Model 2 adjusted for sex, age, marital status, educational status, occupational status, and physical activity level.

## Discussion

The study suggested two major dietary patterns (westernized and traditional), which are significantly associated with metabolic risk factors such as hypertension.

Limited studies are available investigating dietary patterns and associated health outcomes in African adults. A study on urban people in Ghana^32^, identified two types of dietary patterns: ‘purchase pattern’ characterized by high intakes of sweets and sweet drinks, rice, foods rich in protein (red meat, poultry, egg, milk), plant oils (vegetable oil and margarine), fruits and vegetables (carrot, lettuce, cucumber), and low intakes of plantain); and ‘traditional pattern’ characterized by high intakes of plantain, green leafy vegetables, beans, garden eggs, fish, maize, palm oil, okra, and fruits. A study in Cameroon^33^, on the other hand, identified two dietary patterns: ‘fruits and vegetable pattern’ (heavily weighed by fresh fruits and green and dark yellow vegetables, tubers, and legumes) and ‘meat pattern’ (elevated bush meat, poultry, red meat, with low intake of sweets, cakes, and sugar). Finally, a Tanzania study^35^ identified five dietary patterns: ‘traditional coast’ (fruits, nuts, starchy plants, fish, tea); ‘purchase pattern’ (bread or cakes, sugar, tea), ‘traditional inland’ (cereals, oil/fat, vegetables, ); ‘pulses pattern’ (pulses, vegetables); and ‘animal products’. In addition to being inconsistent in the number of dietary patterns derived, these studies differ from our findings in that fruit and vegetable foods were loaded with traditional dietary patterns. However, our study loaded them high in the westernized dietary pattern with meat-based foods. This could be due to variations in food insecurity, availability, affordability, food cultures/taboos, agricultures, and economies between the countries^36–39^. Our findings also differ from dietary patterns identified in studies targeting Lebanon^40^, Mexican^17^ and Brazilian adults^41^. The study from Lebanon found ‘Western pattern’ (potato, grains, soda, sweets, pizza, meat, fast foods, poultry, fat/oils, and nuts), ‘traditional pattern’ (fruits, vegetables, legumes, olives, whole bread, hot drinks, dried foods, crushed wheat, starchy vegetables, eggs), and ‘fish pattern’ (fish, alcoholic beverage, light soda, low-fat diet products, mayonnaise, breakfast cereals). Whereas the Mexican dietary patterns include^17^: ‘Westernized’ (high intake of refined cereals, snacks, desserts, sweets and sugar, pastries, soda), high animal fat and protein (eggs, poultry, red meats, sausages, and alcohol), and ‘prudent’ (high consumption of vegetables, legumes, nuts and seeds, fruits and whole grains). A study of Brazilian adults also identified the following patterns^41^:‘Western’ (pizza/pasta, stroganoff, hamburgers, sweets, chocolate, soft drinks, processed meat), ‘snacks and processed foods’ (cheese, cream cheese, snacks toasted, natural spices, alcohol, olive oils/oils), ‘healthy’ (breakfast cereals, semi and skim milk, yogurt and fermented milk, vegetable, fruits, natural fruit juices), ‘traditional Brazilian’ (meat red and pork, beans, rice and roots, caffeinated beverage). Although there are some common characteristics in the type of foods categorized in the westernized dietary patterns and the traditional dietary patterns with the previous studies, the variations across these studies show the difficulty of establishing a common dietary pattern applicable to other settings, such as sub–Saharan Africans, as they are in the early stage of nutritional transition^36,42^.

This study showed that young adult age, being married, and having good income are significantly associated with frequent consumption of westernized dietary patterns. This finding is consistent with the dietary purchase pattern in Ghana^32^, and the meat and sweet pattern in high income Filipino adults^43^. In Ethiopia, westernized pattern diets such as meats, sweets, and fast and package foods are expensive and can only be afforded by people with good socioeconomic status^44–46^. Healthy food perception in the population might affect diet behavior; many people do not emphasize healthy food options, and some even refer to them as poor people’s diets^47^. This could be because adults in these categories can only afford low-cost foods. It is evident that low-income adults can only afford locally available foods such as cereal-based foods commonly called '*injera with shiro wot,' *although it is the most consumed food type in all categories of the population^47,48^.

Metabolic risk factors such as hypertension are the leading risk factors for the rising burden of NCDs, such as CVD, ischemic heart diseases, stroke, diabetes, and cancer^2,49^. The prevalence of hypertension has been increasing in developing countries, particularly in sub-Saharan Africa, partly attributed to unhealthy dietary habits: high dietary salt intake, and inadequate intakes of fruit and vegetable^3^. In Ethiopia, although some studies reported up to a 40% prevalence of hypertension, a recent systematic review of metabolic risk factors and the current study reveals that one in five (21%) adults are hypertensive^21^. Hypertension increases the incidence of chronic diseases unless prevention and control programs such as screening, identification, and treatment of cases and lifestyle interventions are strengthened^50,51^. This study reveals that the higher quantiles of westernized dietary patterns are significantly associated with reduced hypertension prevalence. Higher intakes of meat/meat-based foods increase the risk of hypertension, while adequate fruit and vegetable intakes are preventive^15,40^. Dietary pattern analysis in Lebanese adults showed that westernized foods, which mainly consist of high intakes of sweets, pizza, meat, fast foods, poultry, and fat/oils, are associated with increased odds of hypertension^40^. High intakes of fats and sweet foods are precursors for metabolic risk factors such as hypertension, obesity, and impaired glucose tolerance; thus, westernized dietary patterns that include fast foods, fish, and sweet foods, fats or oil foods are linked with higher readings of systolic and diastolic blood pressure measures^52^. In the current study, however, preventive foods such as fruits and risky foods like meat-based foods loaded high in the same group, in the westernized pattern. This could be because, in resource-limited countries like Ethiopia, food preference and consumption are associated with the income of people; fruits/vegetables and meat/fatty foods are mainly consumed by adults who have good income as they are unaffordable to the marginal and poor people. The intake of risky meat and fatty foods could be offset by the protective effect of healthy foods like fruits. The early stage of nutrition transitions in the population could explain this variation; people tend to consume primarily local plant-based foods, but as their income improves, they start to consume processed and refined foods high in fat, sugar, and salt. Although the relationship between income and nutrition is complex, in the earliest stage, the transition has a positive impact in improving overall health and reducing risk of chronic diseases by increasing access to diversified and nutritious foods and increase the ability to purchases foods like fruit/vegetable, lean meats, and whole grain^53,54^.

Studies have shown that westernized dietary patterns are associated with a higher rate of overweight and obesity^17,18^. This research, however, did not show a significant association between westernized dietary patterns and the prevalence of overweight and obesity, including abdominal obesity. In addition, the traditional dietary pattern in this study did not show association with the prevalence of metabolic risk factors. This could be in low-resource settings like Ethiopia; foods like fruits and meat are mainly consumed by people having good incomes as they are unaffordable to low-income people, while traditional diets such as cereal-based foods are followed by the majority of the population. Although this dietary pattern did not show a significant association with the prevalence of metabolic risk factors, it is thought to be related to the low dietary diversity or the monotonous dietary habit in the population, so called '*injera with shiro wot,' *which could increase the risk of chronic diseases.

### Strengths and limitations

This study describes the dietary patterns of adults in Ethiopia and their association with chronic health outcomes like hypertension. Using the PCA method to derive dietary patterns provided a comprehensive characterization of participant's dietary patterns. Nevertheless, the study holds several limitations: (1) dietary frequencies were collected through interviews of adults which might be subject to recall bias, (2) portion sizes were not collected which limits further analysis to examine to the effect individual dietary intake on metabolic risk factors, (3) the study only used a posteriori derived dietary pattern which has no trans-fat related information, (4) the determination of the number of factors and naming of the identified factors (dietary patterns) might have involved subjective decision; however, we have reviewed several similar literatures to classify and interpret the findings consistently, (5) the cross-sectional nature of the study limits causal inference between dietary patterns and metabolic risk factors and it is difficult to rule out the possibility of reverse causations, and finally (6) this study was based on information from adults in Bahir Dar thus generalization to adults in other regions is limited.

## Conclusion

This study suggested two major dietary patterns, westernized and traditional, among adults in Bahir Dar Northwest Ethiopia. Metabolic risk factors like hypertension are significantly associated with higher quantiles, third and fourth of westernized dietary pattern scores. However, in relation to the stage of nutrition transition and socioeconomic conditions, the derived traditional dietary pattern in this study did not show a significant association with the rates of overweight/obesity and abdominal obesity. Identifying major dietary patterns in the population can be informative for dietary interventions in preventing and controlling metabolic risk factors. Further large-scale studies involving adults from different regions of the country are required to corroborate these findings.

### Supplementary Information

Supplementary Information 1.

Supplementary Information 2.

## Data Availability

All data relevant to this study are included in this manuscript.
