# Supplementary material for: Dietary patterns and associations with metabolic risk factors for non-communicable disease
Source: Sci Rep. 2023 Nov 29;13:21028. doi: 10.1038/s41598-023-47548-0 (PMC10687098; doi:10.1038/s41598-023-47548-0)
Supplement: Supplementary file 2 — Supplementary Information 2. [file 41598_2023_47548_MOESM2_ESM.docx]

**Supplementary tables**

Supplementary Table 1: Adjusted odds ratio (AOR) and 95% confidence intervals for westernized dietary patterns

| Variables | Variable  category | Westernized dietary patterns | |  | | | |
| --- | --- | --- | --- | --- | --- | --- | --- |
|  |  | Lowest (Q1)  n (%) | Highest (Q4)  n (%) | AOR | 95% CI | | p-value |
| Age | 18-40 | 39 (43.3) | 51 (56.7) | 5.87 | 2.12 | 16.27 | 0.01 |
|  | 41-55 | 34 (44.7) | 42 (55.3) | 2.44 | 0.96 | 6.16 | 0.06 |
|  | 56+ | 30 (73.2) | 11 (26.8) | Ref |  |  |  |
| Marital status | Married | 45 (40.9) | 65 (59.1) | 2.28 | 1.09 | 4.78 | 0.03 |
|  | Unmarried | 58 (59.8) | 39 (40.2) | Ref |  |  |  |
| Educational status | Primary/lower | 30 (63.8) | 17 (36.2) | 0.83 | 0.31 | 2.23 | 0.71 |
|  | Secondary | 22 (44.9) | 27 (55.1) | 0.99 | 0.44 | 2.24 | 0.98 |
|  | Post-secondary | 51 (45.9) | 60 (54.1) | Ref |  |  |  |
| Occupational status | Securely employed | 30 (47.6) | 33 (52.4) | 0.46 | 0.19 | 1.11 | 0.08 |
|  | Insecure jobs | 41 (57.7) | 30 (42.3) | 0.54 | 0.23 | 1.28 | 0.16 |
|  | Unemployed | 32 (43.8) | 41 (56.2) | Ref |  |  |  |
| Income | Very low | 18 (64.3) | 10 (35.7) | Ref |  |  |  |
|  | Low | 35 (57.4) | 26 (42.6) | 1.41 | 0.52 | 3.82 | 0.49 |
|  | Middle | 39 (38.2) | 63 (61.8) | 3.06 | 1.15 | 8.12 | 0.02 |

**Keys:** Ref: reference; AOR: adjusted odds ratio; CI: confidence interval

Supplementary Table 2: Adjusted odds ratio (AOR) and 95% confidence intervals for traditional dietary patterns

| Variables | Variable  category | Traditional dietary patterns | |  | | | |
| --- | --- | --- | --- | --- | --- | --- | --- |
|  |  | Lowest (Q1)  n (%) | Highest (Q4)  n (%) | AOR | 95% CI | | p-value |
| Age | 18-40 | 49 (55.1) | 40 (44.9) | 0.15 | 0.05 | 0.39 | 0.00 |
|  | 41-55 | 42 (55.3) | 34 (44.7) | 0.350 | 0.14 | 0.86 | 0.02 |
|  | 56+ | 10 (25.0) | 30 (75.0) | Ref |  |  |  |
| Marital status | Married | 65 (59.1) | 45 (40.9) | 0.385 | 0.18 | 0.81 | 0.02 |
|  | Unmarried | 36 (37.9) | 59 (62.1) | Ref |  |  |  |
| Occupational status | Securely employed | 32 (51.6) | 30 (48.4) | 1.844 | 0.82 | 4.149 | 0.13 |
|  | Insecure jobs | 29 (40.8) | 42 (59.2) | 1.651 | 0.72 | 3.770 | 0.23 |
|  | Unemployed | 40 (55.6) | 32 (44.4) | Ref |  |  |  |
| Income | Very low | 10 (35.7) | 18 (64.3) | 2.715 | 1.05 | 7.008 | 0.04 |
|  | Low | 26 (42.6) | 35 (57.4) | 2.040 | 0.97 | 4.263 | 0.06 |
|  | Middle | 60 (60.0) | 40 (40.0) | Ref |  |  |  |
| Physical activity | Active | 68 (49.3) | 70 (50.7) | 1.290 | 0.62 | 2.660 | 0.49 |
|  | Inactive | 33 (49.3) | 34 (50.7) | Ref |  |  |  |

**Keys:** Ref: reference; AOR: adjusted odds ratio; CI: confidence interval.
